# Contribution of anterior cingulate cortex and descending pain inhibitory system to analgesic effect of lemon odor in mice

**DOI:** 10.1186/1744-8069-10-14

**Published:** 2014-02-20

**Authors:** Hiroshi Ikeda, Syuntaro Takasu, Kazuyuki Murase

**Affiliations:** 1 Department of Human and Artificial Intelligence Systems, Graduate School of Engineering, and Research and Education Program for Life Science, University of Fukui, 3-9-1 Bunkyo, 910-8507 Fukui, Japan

**Keywords:** c-Fos, Aromatherapy, Emotion, Formalin test, Anterior cingulate cortex, Lemon oil

## Abstract

**Background:**

Affections are thought to regulate pain perception through the descending pain inhibitory system in the central nervous system. In this study, we examined in mice the affective change by inhalation of the lemon oil, which is well used for aromatherapy, and the effect of lemon odor on pain sensation. We also examined the anterior cingulate cortex (ACC) and descending pain inhibitory system to such regulation of pain.

**Results:**

In the elevated plus maze, the time spent in the open arms was increased by inhalation of lemon oil. The pain behavior induced by injection of formalin into the hind paw was decreased. By inhalation of lemon oil, the number of c-Fos expression by formalin injection was significantly increased in the ACC, periaqueductal grey (PAG), nucleu raphe magnus (NRM) and locus ceruleus, and decreased in the spinal dorsal horn (SDH). The destruction of the ACC with ibotenic acid led to prevent the decrease of formalin-evoked nocifensive behavior in mice exposed to lemon oil. In these mice, the change of formalin-induced c-Fos expression in the ACC, lateral PAG, NRM and SDH by lemon odor was also prevented. Antagonize of dopamine D1 receptor in the ACC prevented to the analgesic effect of lemon oil.

**Conclusions:**

These results suggest that the analgesic effect of lemon oil is induced by dopamine-related activation of ACC and the descending pain inhibitory system.

## Background

Affections have a significant impact on pain perception [1]. Both positive and negative affective states are capable of significantly altering pain sensation. A positive affective state increases the pain tolerance whereas a negative affective state, such as viewing an unpleasant picture with pain cues, decreases the tolerance [2]. In rodents with nerve injury or peripheral inflammation, a tonic aversion to noxious stimuli was revealed by the analgesics-induced conditioned place preference, a procedure commonly used to assess the affective component of pain [3,4].

The use of essential oils can have significant effects on both clinical and experimental pain. For example, cancer pain and the associated anxiety are alleviated by exposure to lavender aroma [5]. Marchand's group has shown that odors can affect pain perception in a gender-related manner [6]. Oil extracted from *Cymbopogon citratus* leaves or from the plants *Croton cajucara* and *Nepeta italica*, increases the reaction time to thermal stimuli and decrease the acetic acid-induced writhing in mice after oral, or intraperitoneal administration [7-9]. In the formalin test, *C. citratus* oil preferentially inhibited the second phase of the response after oral or intraperitoneal administration in mice [9].

ACC neurons are activated by painful stimulation and are involved in affective responses to pain [10,11]. By using formalin-induced conditioned place avoidance (F-CPA) test, the rats with F-CPA produced rigorous emotion-like behaviors and enhanced c-fos expression bilaterally in the ACC [12]. Destruction of neurons originating from the rostral ACC reduced F-CPA without reducing formalin-induced acute nociceptive behaviors in rodents [13,14]. In humans, positron emission tomography revealed that the peripheral noxious stimulus increases neural activity in the ACC [15], and significant changes in pain- evoked activity within ACC consistent with the encoding of perceived unpleasantness [16].

Donahue et al. reported that electrolytic lesion of the ACC decreased paw licking in the formalin test, but did not alter the mechanical hypersensitivity in the L5 spinal nerve ligation model of neuropathic pain [17]. Electrical stimulation to cingulum bundle and surrounding cortical tissue (including ACC) increased hot plate and tail-flick latencies and inhibited formalin-induced pain responses [18]. Short-term ACC activation with electrical stimulation generates long-term inhibitory effect lasting for several minutes on response of wide dynamic range neurons in the spinal dorsal horn to the noxious mechanical stimulation in *in vivo* experiment [19]. These results suggest that the ACC is one neuronal substrate of pain inhibition descending from brain to the spinal dorsal horn.

The essential oil extracted from citrus lemon (lemon oil) was found to induce various behavioral responses in both humans and rodents. In rats, it was found to decrease the stress-induced behavioral effects and to decrease the pentobarbital sleeping time [20,21]. In mice, it has been reported that lemon oil has antidepressant-like effects via the dopmainergic system [22]. In this study, we examined whether or not the inhalation of lemon oil has influence on emotion and pain sensitivity in mice. We also examined the contribution of ACC and descending pain inhibitory system in the central nervous system to the regulation of pain by lemon odor.

## Results

### Effect of lemon oil on the affection

**Figure 1:**
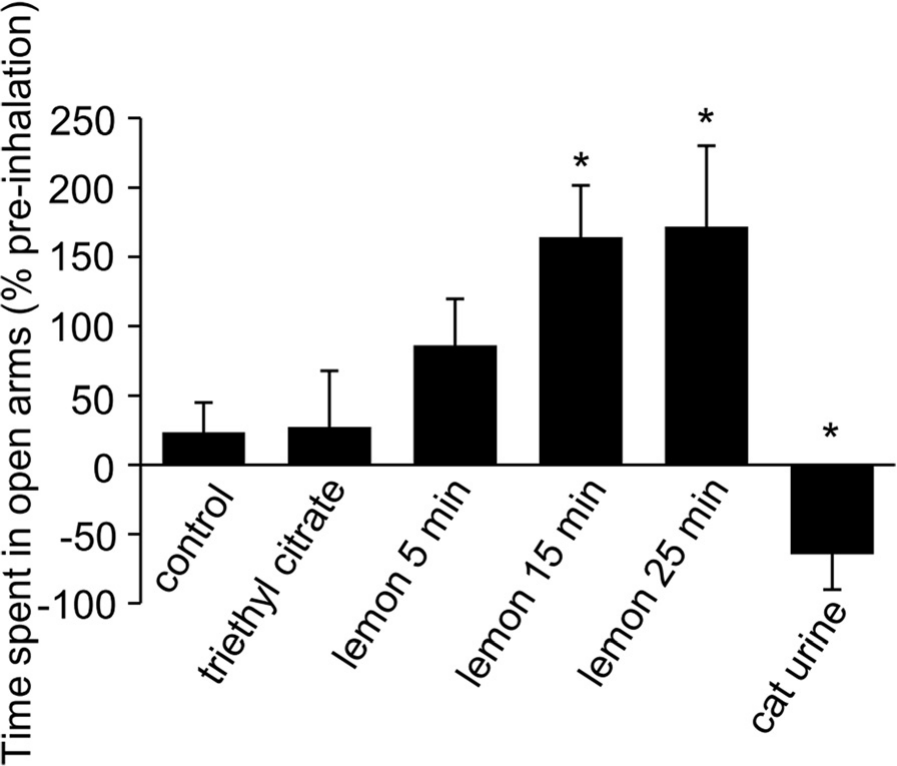
**The Effect of the inhalation of lemon oil on the elevated plus-maze task.** The graph represents the total time spent on the open arms during a 5 min period in control mice, mice exposed to the triethyl citrate, mice exposed to the lemon oil and mice exposed to the cat urine. Each value represents the mean ± S.E. *P < 0.05 compared to the control group.

Before we examine the influence of lemon oil on pain sensitivity, we confirmed the inhalation of lemon oil influences the anxiety-related behavior with elevated-plus maze test without formalin-induced task. The time spent in the open arms was increased in mice exposed to the lemon oil in comparison to control mice and mice exposed to the triethyl citrate (Figure 1 control; 36 ± 19%, triethyl citrate; 27 ± 16%, lemon oil for 5 min; 86 ± 37%, lemon oil for 15 min; 164 ± 34%, lemon oil for 25 min; 171 ± 58% n = 5, p < 0.05). Because exposing to lemon oil for 15 min was enough time to affect the emotion, we apply the odor to mice for 15 min in the following experiments. Additionally, we examined the influence of cat urine as a control odor. In mice exposed to the cat urine, the time spent in the open arms was decreased (Figure 1, -64 ± 25%, n = 7, p < 0.05).

### Effect of lemon oil on the inflammatory pain

**Figure 2:**
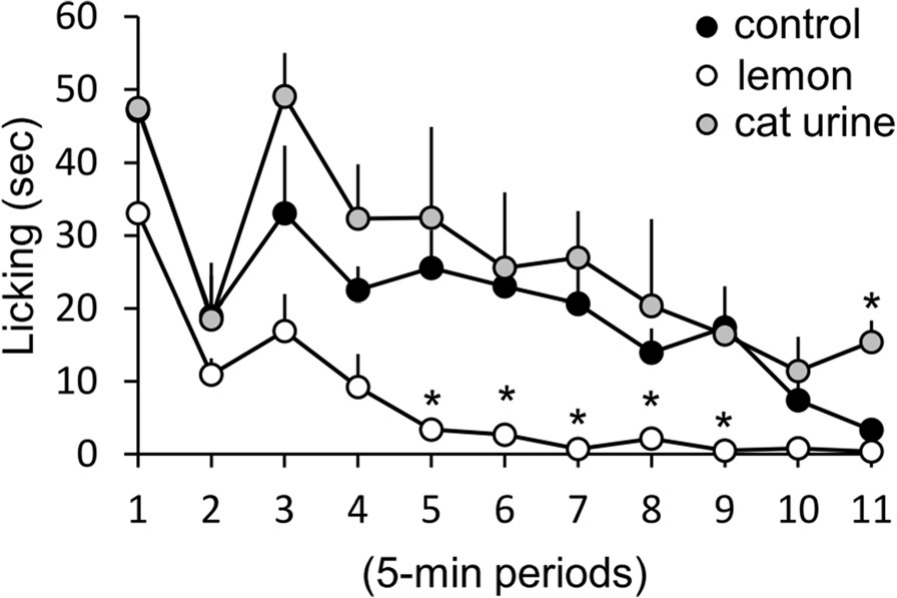
**The Effect of the inhalation of lemon oil on the formalin test.** The graph represents the licking duration time during each 5 min period. Black circles, white circles and gray circles indicate the result of control mice, mice exposed to lemon oil and mice exposed to the cat urine respectively. Each value represents the mean ± S.E. *P < 0.05 compared to the control group.

We examined the effect of inhalation of lemon oil on nociceptive behavior induced by formalin s.c. injection. After exposing in a cage including lemon oil for one hour, mice were injected subcutaneuoly with formalin into the left hind paw. Licking of the injected paw in the cage including lemon oil was recorded for 55 min. The mice exposed to lemon oil significantly decreased the time of licking of injected paw during second phase, but not during the first phase of formalin test (Figure 2, control mice: n = 9, mice exposed to lemon oil: n = 6). The exposure to the cat urine had any effect on the time of licking induced by formalin injection (Figure 1, n = 4).

### Effect of lemon oil on the c-Fos expression

**Figure 3:**
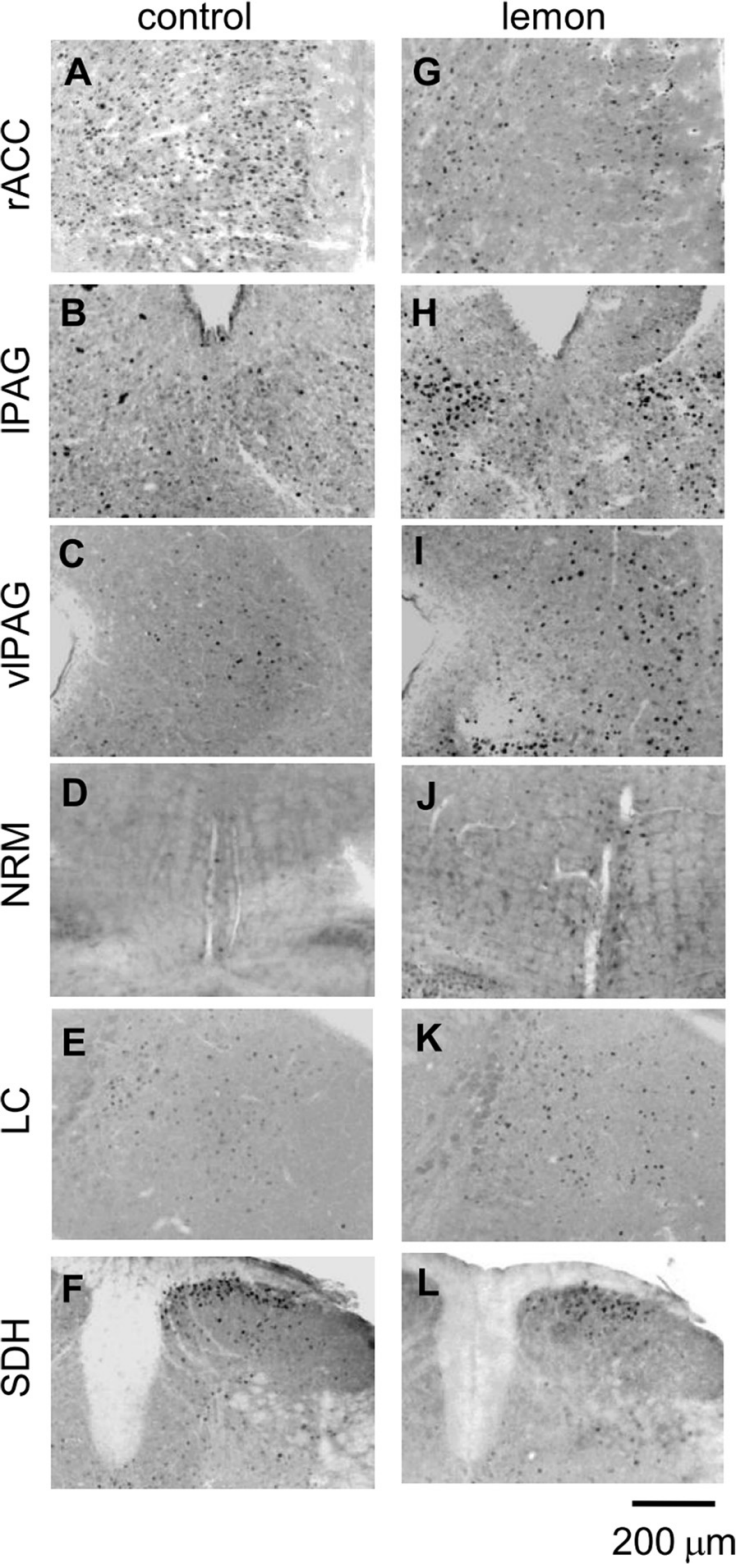
**Photomicrographs showing representative examples of c-Fos immunoreactivity in the rostral anterior cingulate cortex (rACC, A), lateral periaqueductal grey (lPAG, B), ventrolateral periaqueductal gray (vlPAG, C), nucleus raphe magnus (NRM, D), locus coeruleus (LC, E), and the lumbar spinal dorsal horn (SDH, F) of control mice and mice exposed to lemon oil (rACC: G, lPAG: H, vlPAG: I, NRM: J, LC: K, SDH, L).** Black dots indicate the c-Fos expression. Scale bar = 200 μm.

**Figure 4:**
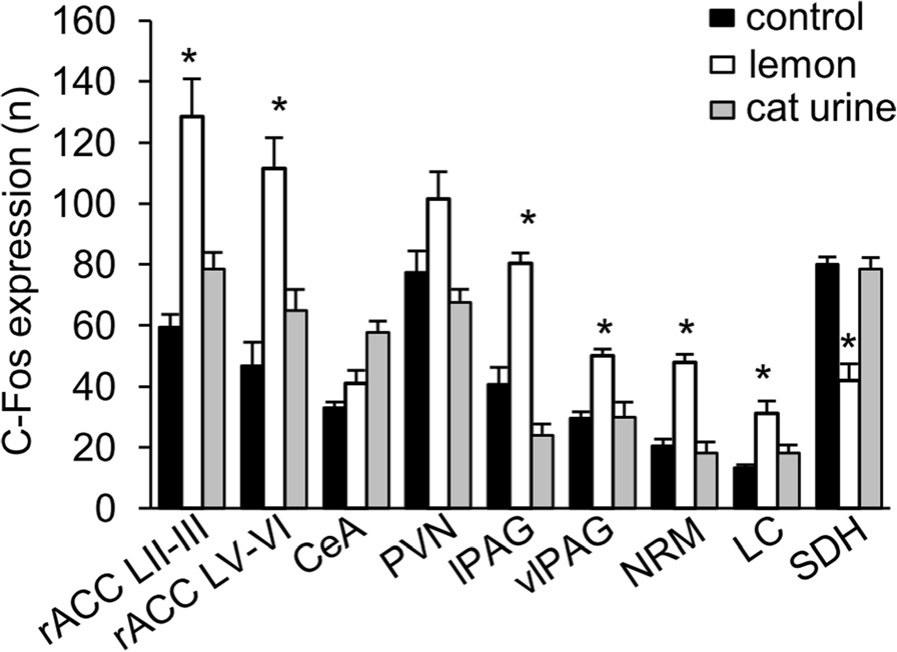
**The number of c-Fos-immuoreactive neurons.** The mean number of c-Fos-immuoreactive neurons in response to injection of formalin in lamina II-III (L II-III) and lamina V-VI (L V-VI) of the rostral anterior cingulate cortex (rACC), the central nucleus of amygdale (CeA), the paraventricular nucleus of hypothalamus (PVN), the lateral PAG (lPAG), the ventrolateral periaqueductal gray (vlPAG), the nucleus raphe magnus (NRM), the locus coeruleus (LC) and the lumbar spinal dorsal horn (SDH). Black, white and gray bars indicate the result of control mice, mice exposed to lemon oil and mice exposed to the cat urine respectively. Each value represents the mean ± S.E. *P < 0.05 compared to the control group.

The c-Fos expression induced by formalin subcutaneuo injection was measured in the ACC and central nucleus of amygdala (CeA) that contribute to affective component of pain, and the hypothalamic paraventricular nucleus (PVN), the periaqueductal grey (PAG), the nucleus raphe magnus (NRM) and the locus coeruleus (LC), which are known as the brain regions related to the descending pain inhibitory system, and the spinal dorsal horn (SDH). The number of c-Fos expression induced by formalin in the lamina II-III (L II-III) and lamina V-VI (L V-VI) of ACC (129 ± 12 vs. 59 ± 4, n = 5, P < 0.05, 112 ± 10 vs. 47 ± 8, n = 5, P < 0.05, respectively), the ventrolateral PAG (vlPAG) (50 ± 2 vs. 29 ± 2, n = 5, P = 0.0004), the lateral PAG (lPAG) (80 ± 3 vs. 40 ± 6, n = 5, P = 0.01), the NRM (48 ± 3 vs. 20 ± 2, n = 5, P = 0.00003) and the LC (31 ± 4 vs. 13 ± 1, n = 5, P = 0.01), but not in the CeA (41 ± 4 vs. 29 ± 2, n = 5, P = 0.05) and the PVN (101 ± 9 vs. 77 ± 7, n = 5, P = 0.06), was significantly higher in lemon oil-treated mice than control mice which was exposed to the triethyl citrate (Figures 3 and 4). In contrast, the number of c-Fos in the SDH was decreased in lemon oil treated mice (Figure 3 and 4, 42 ± 5 vs. 80 ± 2, n = 5, P = 0.002). In mice exposed to the cat urine, the number of c-Fos expression induced by formalin was increased in CeA, but was not changed in other brain regions (Figure 4, CeA 58 ± 4; ACC LII-III 79 ± 5; ACC LV-VI 65 ± 7; PVN 68 ± 4; lPAG 24 ± 4; vlPAG 30 ± 5; NRM 18 ± 4; LC 18 ± 3; SDH 78 ± 4, n =4).

### The effect of ACC destruction on pain behavior and c-Fos expression

**Figure 5:**
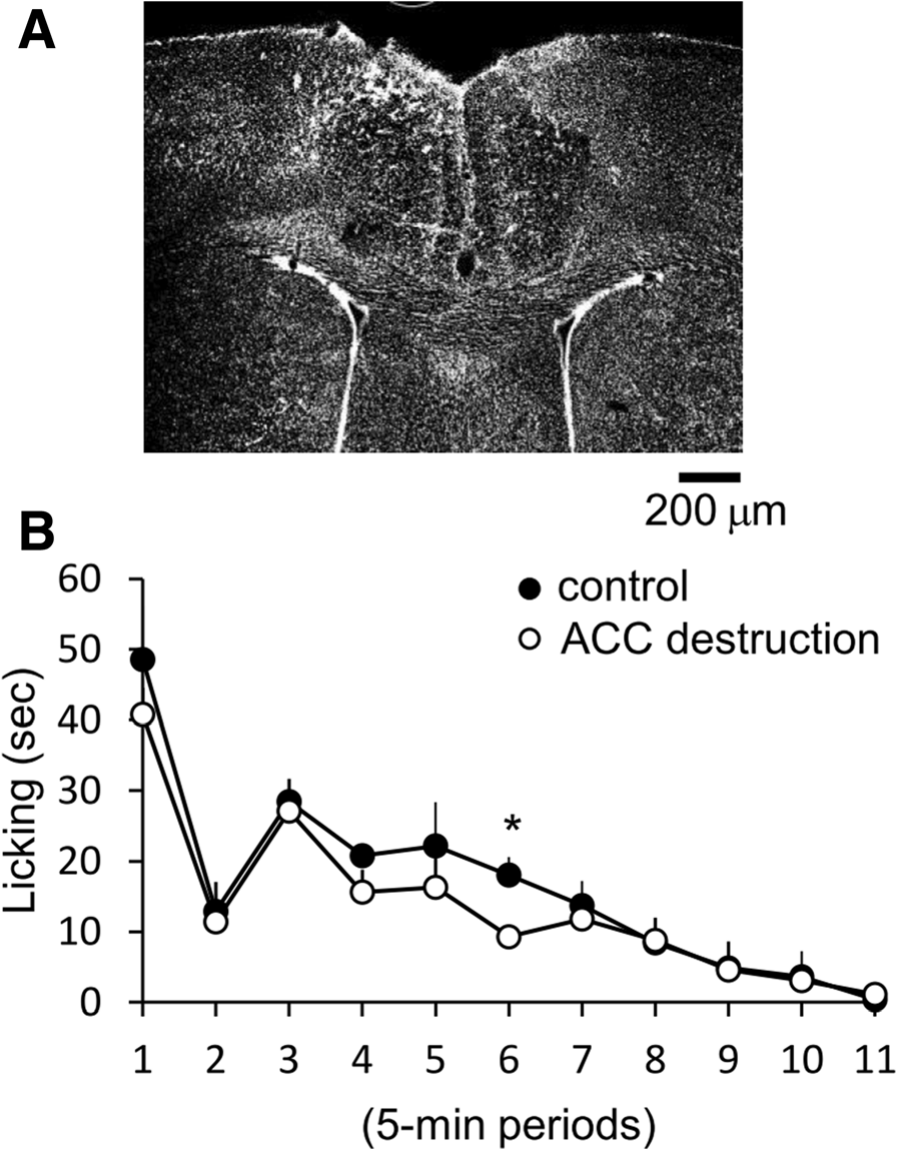
**The Effect of the inhalation of lemon oil on the formalin test in mice destructed the rACC with ibotenic acid. A,** Photomicrograph of representative coronal sections of rACC taken from mice destructed the rACC with ibotenic acid. Section was stained with NeuroTrace. **B,** The graph represent the licking duration time during each 5 min period on the formalin test in mice exposed to lemon oil. Black and white circles indicate the result of control mice and mice destructed the ACC with ibotenic acid respectively. Each value represents the mean ± S.E. *P < 0.05 compared to the control group.

To confirm the contribution of ACC to analgesic effect of lemon oil, we performed destruction of the ACC with ibotenic acid. To examine the effect of destruction on the locomotion, we first examined the frequency of crossing the segmentation lines drown on the floor by open field apparatus. The frequency of crossing in destructed mice was same in comparison to that in control mice which was not injected ibotenic acid (37 ± 2 times/min vs. 39 ± 2 times/min for 5 min period). Then, we examined the effect of destruction on inhibition of pain behavior induced by formalin s.c. injection in lemon oil-treated mice. At one week after destruction of ACC, the exposing to lemon oil failed to decrease the flinching frequency and the time of licking of injected paw during second phase in comparison to that in control mice which was also injected ibotenic acid but was exposed to the triethyl citrate (Figure 5, n = 5).

**Figure 6:**
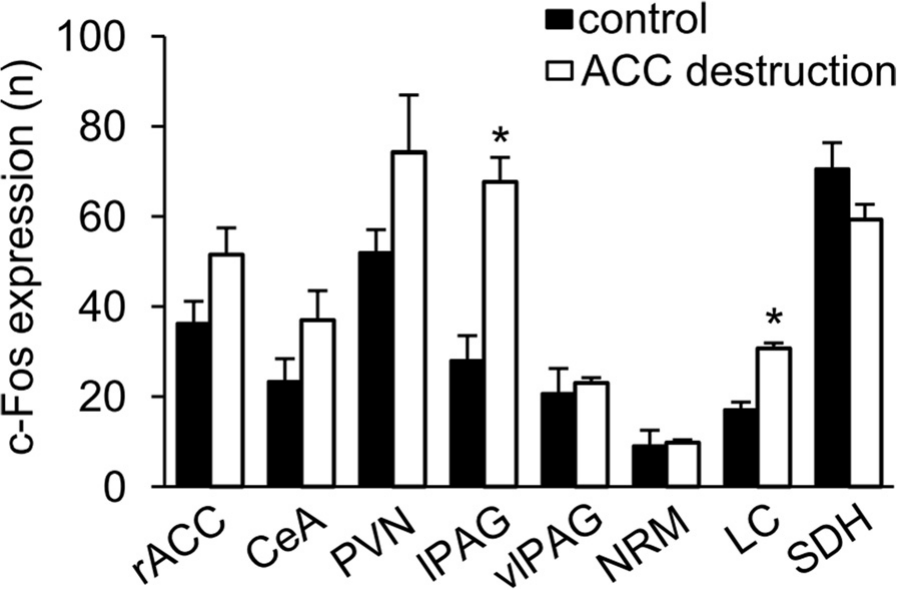
**The number of c-Fos-immuoreactive neurons in mice destructed the ACC with ibotenic acid.** The mean number of c-Fos-immuoreactive neurons in response to injection of formalin in mice destructed the ACC with ibotenic acid in the rostral anterior cingulate cortex (rACC), the central nucleus of amygdale (CeA), the paraventricular nucleus of hypothalamus (PVN), the lateral PAG (lPAG), the ventrolateral periaqueductal gray (vlPAG), the nucleus raphe magnus (NRM), the locus coeruleus (LC) and the lumbar spinal dorsal horn (SDH). Filled and open bars indicate the result of control mice and mice exposed to lemon oil respectively. Each value represents the mean ± S.E. *P < 0.05 compared to the control group.

The number of c-Fos expression induced by formalin s.c. injection in the ACC (Figure 6, 52 ± 6 vs. 36 ± 5, n = 4, P = 0.08), the PVN (Figure 6, 74 ± 13 vs. 52 ± 5, n = 4, P = 0.08), vlPAG (Figure 6, 21 ± 2 vs. 19 ± 3, n = 4, P = 0.35) and NRM (Figure 6, 10 ± 1 vs. 10 ± 3, n = 4, P = 0.43) was not significantly increased by lemon oil in mice with destructed ACC in comparison to control mice which was also injected ibotenic acid but was exposed to the triethyl citrate. However, c-Fos expression in the lPAG (Figure 6, 68 ± 4 vs. 28 ± 4, n = 4, P = 0.003) and the LC (Figure 6, 35 ± 3 vs. 16 ± 2, n = 4, P = 0.001) was still increased by lemon oil in ACC destructed mice. The decrease of c-Fos expression in SDH by lemon oil was partially blocked by the destruction of ACC, but the number of c-Fos was not identical to lemon oil-untreated control mice (Figure 6, 64 ± 4 vs. 70 ± 5, n = 4, P = 0.08).

### The effect of dopamine receptor antagonist on pain behavior

**Figure 7:**
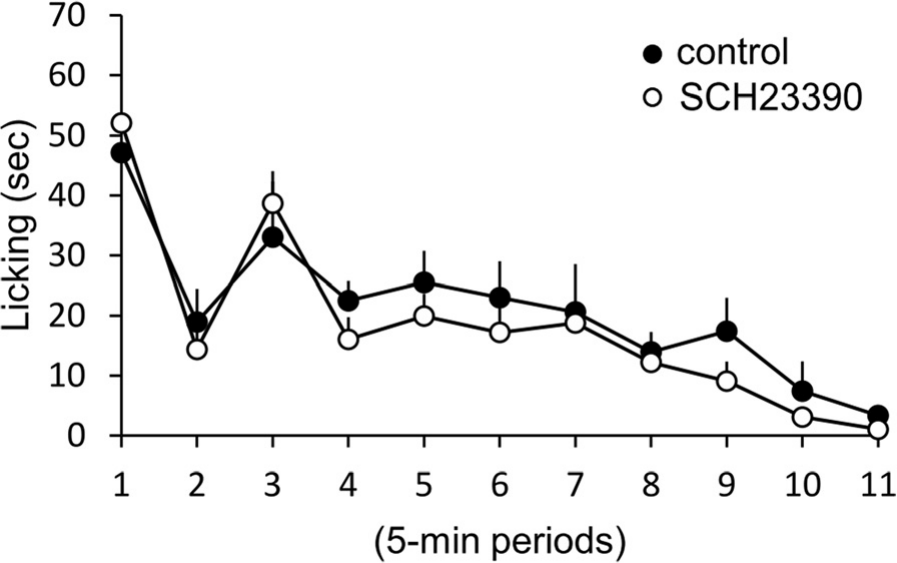
**The Effect of the inhalation of lemon oil on the formalin test in mice injected a domapine receptor antagonist into the rACC.** The graph represent the licking duration time during each 5 min period on the formalin test in mice exposed to lemon oil. Black and white circles indicate the result of control mice and mice injected a domapine receptor antagonist SC23390 into the rACC respectively. Each value represents the mean ± S.E. *P < 0.05 compared to the control group.

To examine the contribution of dopamine in the ACC to pain behavior induced by formalin s.c. injection, dopamine D1 receptor antagonist SCH23390 was microinjected into the rACC. In mice injected the SCH23390 into the rACC, the exposing to lemon oil did not decrease the time of licking of injected paw in comparison to that in control mice which was not injected SCH23390 and exposed to the triethyl citrate (Figure 7, n =4).

## Discussion

In the present study, we have demonstrated that lemon oil decreased the negative affection with the behavioral study in mice. We also showed that the pain behavior induced by formalin s.c. injection was decreased by lemon oil. The c-Fos expression by formalin s.c. injection was increased by lemon oil in ACC, vlPAG, lPAG, NRM and LC, and was decreased in SDH. In mice with destructed ACC, lemon oil did not decrease the pain behavior, and the increase of number of c-Fos expression in the ACC, vlPAG and NRM, and the decrease in the SDH was not induced. In addition, Antagonize of dopamine D1 receptor in the ACC failed to the decrease of pain behavior by lemon oil.

### The effect of lemon odor on negative affection

The essential oil extracted from citrus lemon (lemon essential oil) was found to induce various behavioral responses in both humans and rodents. In rats, it has been reported that lemon odor reduced the immobility time and potentiated the imipramine-induced reduction of immobility time in the force swim test [20]. They have also reported that lemon odor decreased locomotor activity in the open field, suggesting its effects to be different from those of psychostimulants but to be similar to those of antidepressants. In mice, it has been reported that lemon oil have antidepressant-like effects with the elevated plus-maze, the forced swim test, and the open field task via the suppression of dopmaine activity related to enhanced serotonergic neurons [22]. In humans, it has been reported that, although the existence or nonexistence of lemon fragrance did not affect their work efficiency in Kraepelin test, in affects the mitigated exhaustion and maintained vigor [23]. In agreement with these previous studies we confirmed that lemon oil appease the negative affection via dopaminergic system in mice.

### The effect of lemon odor on inflammatory pain

In this study, the lemon odor suppressed negative emotion and in such mice, pain behavior was relieved. Such effect of essential oil on clinical and experimental pain has been reported. For example, cancer pain and the associated anxiety are alleviated by exposure to lavender aroma [5]. Marchand's group has shown that exposure to odors which induce pleasant mood reduces pain perception in human [6]. Aloisi’s group has reported that exposure to the lemon essence for 2 weeks increased the pain threshold to the heat stimulation and decreased the pain behavior to formalin s.c. injection in rats [24]. These results agree with our study. However, in contrast to our study, they have shown that expose to lemon essence increase anxiety-like behavior measured by the hole-board test and the elevated-plus maze [24]. Therefore, further studies such as measurement of hormone concentration and the effect of lemon oil on autonomous nervous system are necessary to reveal the analgesic effect of lemon odor is depend on appease of negative affection.

### Contribution of ACC and descending inhibitory pathway to analgesia by lemon oil

In this study, lemon oil facilitated the c-Fos expression induced by formalin s.c. injection in PAG, RVM and LC, and decreased that in the SDH. It is well established that the pain signals from the periphery to the SDH is regulated by descending control from higher centers that originate at the level of the cortex, the thalamus and the brainstem [25]. The descending inhibition from the cortex and the thalamus is, in part, mediated via relay station in the brainstem [25]. Therefore, our results suggest that analgesic effect of lemon odor is mediated by such descending inhibitory system via the PAG, the RVM and the LC.

In this study, c-Fos expression induced by formalin injection was also increased in the ACC by lemon odor and the destruction of ACC inhibited the analgesic effect of lemon odor and suppressed the increase of c-Fos expression by lemon oil in the ACC, lPAG and NRM, but not in vlPAG and LC. Sakabe et al. have reported that the ACC was activated by odor of hexenol/hexenal which is known to have a healing effect on the psychological damage caused by stress, but not by odor of amylacetate or acetic acid [26]. They have suggested that the ACC may be activated by odors that stimulate the limbic system, and hexenol/hexenal may simultaneously activate both the olfactory and limbic systems, which may activate the ACC effectively. In this study, the c-Fos expression in the ACC was increased by lemon oil, but not by cat urine. Taken together, odor of lemon oil may do not activate the ACC directly, but activated via a specific pathway in the limbic system which is activated by lemon oil. Further studies are necessary to reveal how lemon oil works in brain especially the ACC.

A large number of studies have revealed that the ACC neurons are activated by noxious stimulation and are involved in affective responses to pain [10,11]. Hardy et al. have reported that electrical stimulation to singulum bundle and surrounding cortical tissue (including ACC) increased hot plate and tail-flick latencies and inhibited formalin-induced pain responses [18]. Ma et al. have also reported that short-term ACC activation with electrical stimulation generates long-term inhibitory effect lasting for several minutes on response of wide dynamic range neurons in the spinal dorsal horn to the noxious mechanical stimulation [19]. The contribution of ACC to the descending facilitation of pain has also been reported. Zhang et al. have revealed that high-frequency tetanic electrical stimulation of the ACC and microinjection of NMDA or homocysteic acid into the ACC enhanced the C-fibre-evoked field potentials in the spinal dorsal horn and shortened paw withdrawal latencies to noxious heating [27]. Ren et al. have reported that lesion of ACC decreased the bee venom-induced pain hypersensitivity [28]. It is thought that PAG receives direct projections from the ACC [29,30]. Recently, Kong et al. have reported that the presence of ACC-PAG-RVM network using resting fMRI data from human [31]. Fulbright et al. have reported that ACC is activated in response to the odor of sweet citrus by using fMRI imaging in human [32].

## Conclusions

Our results suggest that the analgesic effect of lemon odor is mediated by the descending inhibitory system via PAG-RVM pathway and the origin of this inhibitory system may be the dopamine-related activation of ACC neurons by the lemon odor. In this study, we have not revealed how LC activated by lemon odor and how LC contributes to the analgesic effect of lemon odor. The destruction of other affection-related brain regions such as amygdala or insular cortex may be necessary to make it clear. It is also necessary to reveal the contribution of neurotransmitters which are related to the descending pain inhibition such as opioids, 5-HT and noradrenalin to reveal the complete mechanism of lemon odor-induced analgesia in the future. Although further studies are necessary, the odor-induced analgesia may lead to a novel approach for treating the inflammatory pain.

## Methods

### Animals

All animal studies were undertaken using protocols approved by the University of Fukui animal ethics committee. 5-weeks-old ICR mice were used.

### Inhalation of essential oil

The lemon oil was supplied by Soda Aromatic Co., Ltd. (Tokyo, Japan). Lemon oil was mixed with triethyl citrate (Nacalai Tesque, Kyoto, Japan) and was then soaked up by tissue paper set on the upper side of an inhalation box. In all experiments, 1 ml of the lemon oil was vaporized in the container (30 cm × 23 cm × 15 cm). Triethyl citrate was applied as the control treatment. The inhalation of essential oil was started 5, 15 and 25 min before the behavioral test.

### Elevated plus-maze test

Wooden plus-maze apparatus, elevated to a height of 40 cm, consisted of two open arms (40 × 8 cm) and two closed arms (40 × 8 cm, and 20 cm high walls), arranged so that the two arms of each type were opposite each other. The time spent in open arm during a 5 min period was recorded. The test was performed for two days. At the first day, the test performed without exposing any odor, and at the second day, the test was performed in mice exposed to lemon odor.

### Formalin test

Formalin solution (30 μl, 5% in saline) was injected subcutaneously into the plantar surface of left hindpaw. After formalin injection, the mice were immediately placed in a clear plastic box. The duration of licking and frequency of flexing of the injected paw was recorded in 5-min intervals for 55 minutes after the injection.

### Immunohistchemistry

Mice were deeply anesthetized with an intraperitoneal injection of 2,2,2-tribromoethanol (200–300 mg/kg body weight) and perfused intracardially with 0.01 M PBS followed by 4% cold, buffered paraformaldehyde. The brain and spinal cord was then removed immediately, post-fixed at 4°C overnight in the same fixative and then cryoprotected in 20% sucrose in PBS (pH 7.4) for 48 h and sectioned at 40 μm. Nonspecific antibody binding was inhibited by incubating the slices in 3% normal goat serum. Slices were then incubated for 24 h at 4°C with rabbit anti-c-Fos monoclonal antibody (1:5000; Calbiochem, San Diego, CA, USA). After incubation, tissue sections were washed and incubated for 1 h at room temperature with secondary antibody solution (anti-rabbit IgG-conjugated Alexa Fluor 568, 1:400; Molecular Probes).

Images were captured using a DP72 camera (Olympus, Tokyo, Japan) mounted on an AX80 microscope (Olympus, Tokyo, Japan) and analyzed using the NIH image-based ImageJ 1.43u program (National Institute of Health, USA). To quantify c-Fos immunoreactive nuclei in square fields of each brain region, background staining was subtracted using the same threshold value for each section and particles that met size requirements were counted.

### Distraction of ACC by ibotenic acid

Mice were anesthetized with an intraperitoneal injection of 2,2,2-tribromoethanol (200–300 mg/kg body weight) and were then positioned in a stereotaxic apparatus. A hole was drilled on each side of the rostral ACC area (AP = 1.0, ML = 0.3 from Bregma) through the skull in order to allow insertion of a 1-μl Hamilton syringe needle. Ibotenic acid (0.2 μl each side, Wako Pure Chemical Industries, Osaka, Japan) was injected and the syringe remained in place for 10 min after each injection to prevent the spread of the agent to the surface of the brain. This procedure was then repeated in the opposite hemisphere. The distraction area was confirmed with NeuroTrace (1:200, Molecular Probes). The data from the animals with injection sites outside rostral ACC were excluded. At one week after injection, mice were used for the open field test and the formalin test.

### Open field test

The open field area is made of black acrylic (50 × 50 cm, and 30 cm high walls). The floor had five squares of equal area. Each mouse was placed in the corner of a compartment and ambulation was recorded with a video camera for 5 min. The number of squares crossed with the four paws was counted.

### Application of dopamine receptor antagonist in the ACC

Mice were anesthetized with isoflurane (1%) and were then positioned in a stereotaxic apparatus. A hole was drilled on each side of the rostral ACC area (AP = 1.0, ML = 0.3 from Bregma) through the skull in order to allow insertion of glass pipette Hamilton syringe needle. SCH23390 (0.5 μl each side, ) was injected and the syringe remained in place for 10 min after each injection to prevent the spread of the agent to the surface of the brain. This procedure was then repeated in the opposite hemisphere. The data from the animals with injection sites outside rostral ACC were excluded. At after injection, mice were used for the open field test and the formalin test.

### Statistical analysis

The results are expressed as means ± standard error of the mean (SEM). Student’s t-test was used to assess statistical differences. Statistical significance was indicated by *p < 0.05.

## Competing interests

The authors declare that they have no competing interests.

## Authors’ contributions

HI conceived and designed the study. ST performed experiments and analyzed data. HI and KM wrote the manuscript. All authors have read and approved the final manuscript.
